# The epidemiology of road traffic injury hotspots in Kigali, Rwanda from police data

**DOI:** 10.1186/s12889-016-3359-4

**Published:** 2016-08-02

**Authors:** Anjni Patel, Elizabeth Krebs, Luciano Andrade, Stephen Rulisa, João Ricardo N. Vissoci, Catherine A. Staton

**Affiliations:** 1 Department of Surgery, Division of Emergency Medicine, Duke University, Durham, NC USA; 2 Department of Emergency Medicine, Section of Prehospital and Disaster Medicine, Emory University, Atlanta, GA USA; 3 State University of Maringá, Maringá, Paraná Brazil; 4 Department of Obstetrics & Gynecology, University of Rwanda School of Medicine, Kigali, Rwanda; 5 Department of Medicine, Faculdade Ingá, Maringá, Paraná Brazil; 6 Duke University Medical Center, DUMC Box 3096 2301 Erwin Road, Duke North, Suite 2600, Durham, NC 27710 USA; 7 Division of Global Neurosurgery and Neurosciences, Department of Neurosurgery, Duke University Medical Center, Durham, USA

## Abstract

**Background:**

Road traffic injuries (RTIs) are the eighth-leading cause of death worldwide, with low- and middle-income countries sharing a disproportionate number of fatalities. African countries, like Rwanda, carry a higher burden of these fatalities and with increased economic growth, these numbers are expected to rise. We aim to describe the epidemiology of RTIs in Kigali Province, Rwanda and create a hotspot map of crashes from police data.

**Methods:**

Road traffic crash (RTC) report data from January 1, 2013 to December 31, 2013 was collected from Kigali Traffic Police. In addition to analysis of descriptive data, locations of RTCs were mapped and analyzed through exploratory spatial data analysis to determine hotspots.

**Results:**

A total of 2589 of RTCs were reported with 4689 total victims. The majority of victims were male (94.7 %) with an average age of 35.9 years. Cars were the most frequent vehicle involved (43.8 %), followed by motorcycles (14.5 %). Motorcycles had an increased risk of involvement in grievous crashes and pedestrians and cyclists were more likely to have grievous injuries. The hotspots identified were primarily located along the major roads crossing Kigali and the two busiest downtown areas.

**Conclusions:**

Despite significant headway by the government in RTC prevention, there continue to be high rates of RTIs in Rwanda, specifically with young males and a vulnerable road user population, such as pedestrians and motorcycle users. Improvements in police data and reporting by laypersons could prove valuable for further geographic information system analysis and efforts towards crash prevention and targeting education to motorcycle taxis could help reduce RTIs in a severely affected population.

## Background

Injuries are a major global health problem contributing annually to over 4.5 million deaths worldwide [1]. Approximately 650 million people worldwide are living with disabilities due to injury. Over 90 % of these unintentional injuries and 90 % of disability adjusted life years occur in low and middle-income countries (LMICs) [2, 3]. Nearly 60 % of road traffic deaths occur in individuals between 15 and 44 years old and over 75 % of road traffic deaths occur in males [4].

The number of worldwide road deaths has steadily risen from 1990 to 2013 with the majority of deaths occurring in LMICs and the highest road traffic fatality rate is in Africa [1, 4]. World Bank predictions are that with further economic development and increased motorization, global road traffic deaths will rise by more than 35 % from 2010 to 2020. This international crisis has led to the United Nations General Assembly and the World Health Organization have created a “Decade of Action for Road Safety” with a global plan for addressing road traffic crashes and deaths.

Vulnerable road users (VRUs) such as motorcyclists, pedestrians, and bicyclists bear a disproportionate share of the injury burden [5]. Motorcycles are already ubiquitous and still a rapidly growing form of transport in Africa for personal and commercial transportation. In 2013 Rwanda had more 2- and 3-wheeled registered vehicles than cars [4, 6]. Developing countries, including Rwanda are therefore facing a major public health challenge of VRU injuries [7].

Kigali, the capital city of Rwanda, is home to just over 1 million people [8]. Following the civil unrest and genocide of the 1990s, there has been dramatic socioeconomic development including a large influx of personal and commercial transport utilizing motorcycles [9]. The number of licensed taxicab companies and cooperatives in Rwanda has increased from just 13 in 2011 to 50 in 2014 and public transportation companies more than doubled from 25 to 57 in the same time period. Similarly, increases in the number of licensed minibuses and buses were also seen. Although the number of new licenses issued in all categories has decreased over the last few years, the trend in new licenses issued leans heavily towards males, with a total of 152,595 new licenses issued to males between 2011 and 2014 compared to only 16,077 for females [10]. Estimated road traffic deaths in Rwanda were some of the highest in the world during the genocide. With improved infrastructure and road safety program development by the government, these numbers started to trend downwards, however national data suggests a rising trend in numbers of injured persons and deaths. In 2012, there were 4471 road traffic crashes (RTCs) throughout Rwanda with national statistics indicating 1451 injured persons and 220 deaths. Data from 2015 demonstrates injured persons now number 2508 with 366 deaths [10].

Based on hospital data from Kigali, we know road traffic injury (RTI) patients who seek care are primarily male. (58 %) [11]. In 2008, Twagirayezu studied RTI patients from one hospital with available police data from 2002 to 2005 and found VRUs were involved in 41 % of the RTCs and 56 % of road traffic deaths prior to arriving at the hospital were VRUs. The study also reported a very high proportion of limb injuries (56.3 %), which they attributed to a high number of pedestrian injuries [12].

A promising methodology for injury prevention is geographic information systems (GIS), a tool for spatial analysis, pattern and cluster identification of RTCs [13, 14]. This tool allows for an in-depth built environment analysis of RTC hotspots to identify and suggest the location and, often, type of amenable interventions [15]. GIS and spatial analyses can use pre-existing police, prehospital, hospital or mortality reporting datasets, yet in most LMICs these datasets are inadequate, incomplete or unavailable [3, 4, 16]. While police data has its limitations, it is the currently the most commonly used method of informing spatial analysis for road safety audits as there is no other more complete reliable options. Therefore, this project both describes the epidemiology of RTCs and hotspots for RTCs in Kigali, Rwanda, using police data in order to identify which populations of road users should be the priority for road safety initiatives in Kigali and which locations might warrant further safety interventions.

## Methods

### Study setting

**Fig. 1:**
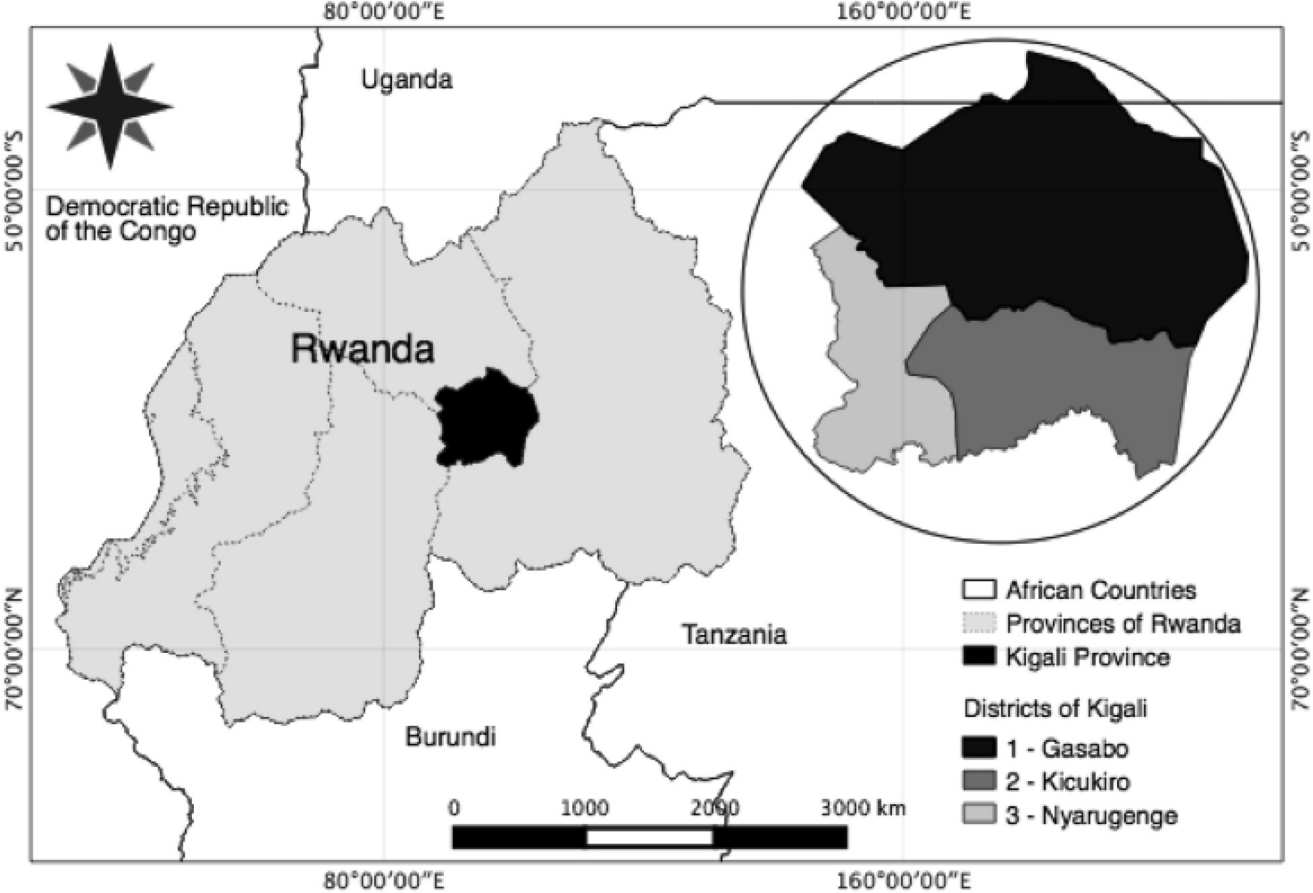
Location of Kigali Province with 3 districts (Nyarugenge, Kicukiro, Gasabo), Republic of Rwanda

Rwanda, a landlocked country in East Africa, is the most densely populated country in the world with over 11 million residents (Fig. 1) [17]. The total population of the capital city of Kigali is about 1.13 million with a mean population density of 1552 people/km^2^ [8]. Kigali is made up of three districts, Gasabo, Kicukiro and Nyarugenge, with a combined mean road density of 0.093 km/km^2^ and unpaved mean road density of 0.12 km/km^2^ [18]. In the past 20 years, Rwanda has achieved remarkable economic development, increasing the per capita gross domestic product of the country from 753 million dollars in 1994 to 7.89 billion dollars in 2014. Rwanda aims to reach middle income country status by the year 2020 [17].

### Ethics

Our study was approved by the Institutional Review Board for Duke University, the Ethics Committee of CHUK hospital, and the National Health Research Council of the Ministry of Health of Rwanda. We obtained formal permission to interview motorcycle taxi drivers from the Rwanda Federation of Transport Cooperative and were granted access to RTC reports by the commissioner of the Rwanda National Police.

### Variables

The primary variable studied was the geographic location of RTIs, as determined by latitude and longitude of the crash point, in the city of Kigali during 2013. Crash severity was categorized according to the most serious injury of any person involved in the crash: damage only (no injuries, vehicle damage only), non-grievous injury, grievous injury (could cause disability or death), and fatal crashes (one or more persons died due to the crash). This categorization was taken directly from police records. The severity of the crash was dichotomized into non-grievous (no injuries and non-grievous) and grievous (any grievous or fatal injury) crashes for bivariate and multivariate analyses. We collected information of up to 5 different victims per crash when available. As listed in the police data, vehicle types as well as crash victims were listed as ‘primary’ and ‘secondary’ but these were arbitrary designations by police officers: we continued this labeling in our results. Crash causality was determined by the police officers who conducted the investigation.

Crash characteristics included were day of the crash (weekend or weekday), time of the crash (day, dawn/sunset, night: per police data about lighting at time of the crash), type of crash, primary and secondary crash vehicle types as defined by the police data collection, road visibility, road characteristics (dirt or paved), road conditions (dry or wet). Characteristics of individual crash victims included age and gender. Due data collection limitations, only data for age of drivers was available and not ages for all victims.

### Data source and collection

Data from Kigali Traffic Police was extracted from all accessible records of RTCs that occurred between January 1 and December 31, 2013. Completed paper RTC reports are stored in a central location at the Kigali Traffic Police office in multiple binders and recorded in a register book. Between January 14 and February 17, 2014, trained data collectors analyzed every available paper crash report from 2013 and used tablet devices to record selected data points directly into a secure REDCap (Research Electronic Data Capture) database. Reports were unavailable for a variety of reasons including the RTC being reviewed by the judicial system, parties involved in the RTC failed to file or complete a report, or reports were misplaced. There was no way to determine if missingness was non-random due to limited information from missing reports and in the register book. Data collection was conducted with REDCap electronic data capture tools hosted at Duke University [19]. REDCap is a secure, web-based application designed to support data capture for research studies, providing data entry through an interactive interface [19].

Each RTC police report paperwork was completed by a police officer; and therefore at times, data collectors noted all missing values. Quality assurance of data extraction was performed by an independent reviewer looking for missing or nonsensical responses. When errors in data collection were found, a second data collector reviewed the RTC report in question to correct mistakes. When possible, the precise location of each RTC was determined. If it was impossible to identify the location using data from the RTC police reports, clarification was obtained from a traffic police officer. All instances when it was impossible to determine a precise RTC location were noted.

### Data analysis

Data analysis was conducted in two steps. Initially, step 1 included an epidemiological overview of RTCs in Kigali. Descriptive statistics were reported mainly with frequencies and tabulations. Exploratory graphical analysis was used to evaluate data distribution and quality. Bivariate chi-square and t-test, and multivariate logistic regression models were conducted to evaluate the association between crash characteristics (e.g., road characteristics, time of day) and crash severity (grievous vs. Non-grievous), reported through odds ratios and confidence intervals. Since age and gender are individual level variables, and not crash-level variables, we excluded them from the multivariate analysis. Two variables (weather condition and road condition) had data quality issues with more than 20 % of values missing and with a non-random missingness pattern. All other variables showed a small amount of missing information, so in order to verify its impact on the analysis, we conducted a multiple imputation (MI) model by chained equation through the mice package [20]. The MI model showed no improvement over the initial model, so we chose to exclude missing cases. In step 2, we applied a spatial analysis to describe the geographical distribution of RTCs.

### Spatial analysis

Spatial analysis using a point process framework was used to identify locations with the highest frequency of crashes and the characteristics of crash locations in Kigali’s geographical space.

The georeferenced cartographic database of Kigali administrative districts Gasabo, Kicukiro, and Nyarugenge is freely available online in SHP format (shapefile) at the National Institute of Statistics Rwanda [21]. The spatial distribution of the RTC points using exploratory spatial data analysis (ESDA) allowed us to locate hotspots through map visualization and to evaluate observed patterns, determining if the events showed random characteristics, clusters, or even distribution [22–24]. We utilized the Kernel Density Estimator to create a point density map (raster image) through heatmap plugin in Quantum GIS software, version 2.4 [25]. This estimator is a two-dimensional function that weighs the crashes within a defined radius, indicating higher or lower concentration of events analyzed, so-called hotspots [22, 26]. For this study, we used a bi-weighted kernel; events were weighted by the density of events within 1000 m and by the injury severity.

## Results

A total of 2589 crashes were analyzed with 4689 victims involved, of which 685 were injured and 85 died. Gender, collected for drivers only, was mostly male (92.5 %), and the average age was 36.4 (sd 9.6) years. Fatal/grievous injuries were less frequent in males and happened to younger victims (Table 1). Of all crashes, 18.5 % were considered fatal or grievous. Most RTCs occurred on Mondays, Tuesdays, and Fridays (15.9, 15.1 and 15.3 %, respectively), with the lowest number of crashes occurring on Sundays (12.7 %). Crashes mainly occurred during the day time (65.9 %). The primary data only differentiated time of day into day, dawn, or night. Road visibility was only separated into daylight, streetlight, or dark with the majority of crashes occurred in daylight (65.9 %). Road conditions at crashes were frequently paved (95.6 %) and dry (95.5 %). Crashes were mostly caused by cars (43.8 %) and trucks (26.8 %), followed by buses (14.6 %) and motorcycles (14.5 %). Primary victims of crashes were mainly in cars (53.9 %), followed by motorcycles (15.4 %), trucks (11 %) and pedestrians and cyclists (11.4 %).

**Table 1:** Crash / primary victim factors associated with grievous injury

	Total	Grievous	Non-grievous	OR (IC 95 %)
Age, Mean (SD)	36.41 (10.32)	31.98 (11.96)	36.79 (10.14)*	–
Male, N (%)	4248 (92.5)	380 (86.36)	3749 (93.14)*	–
Time of day, N (%)				
Day	1268 (49.1)	191 (41.1)	1055 (51.1)*	Ref
Dawn	382 (14.8)	89 (19.1)	285 (13.8)	2.41 (1.41;4.10)**
Night	930 (36.1)	185 (39.8)	723 (35.1)	1.69 (1.07;2.66)**
Day of Week, N (%)				
Monday	410 (15.9)	76 (16.3)	319 (15.5)	Ref
Tuesday	388 (15.1)	64 (13.7)	320 (15.6)	1.02 (0.53;1.94)
Wednesday	352 (13.7)	55 (11.8)	293 (14.2)	0.87 (0.44;1.69)
Thursday	354 (13.7)	73 (15.6)	276 (13.4)	0.97 (0.52;1.81)
Friday	394 (15.3)	72 (15.4)	308 (15.0)	1.02 (0.54;1.92
Saturday	351 (13.6)	64 (13.7)	283 (13.6)	1.25 (0.64;2.41)
Sunday	328 (12.7)	63 (13.5)	258 (12.5)	1.04 (0.53;2.03)
Visibility, N (%)				
Daylight	1487 (65.9)	266 (65.4)	1197 (66.0)	Ref
Streetlight	544 (24.1)	96 (23.6)	441 (24.3)	0.79 (0.49;1.27)
Dark	225 (10.0)	45 (11.0)	175 (9.7)	1.01 (0.51;1.94)
Primary Crash Vehicle, N (%)				
Pedestrians & Cyclists	6 (0.3)	3 (0.2)	–	
Cars	1121 (43.8)	157 (34.1)	948 (46.1)*	Ref
Motorcycles	372 (14.5)	143 (31.0)	219 (10.1)	4.27 (2.54;7.24)**
Buses	374 (14.6)	67 (14.5)	301 (14.6)	2.11 (1.22;3.63)**
Trucks	686 (26.8)	91 (19.7)	583 (28.4)	1.88 (1.19;3.00)**
Primary Victim Vehicle, N (%)				
Cars	1214 (53.9)	37 (8.7)	1156 (64.6)*	Ref
Pedestrians & Cyclists	257 (11.4)	220 (51.8)	34 (1.8)	263.64 (147.02;496.53)**
Motorcycles	346 (15.4)	139 (32.8)	196 (11.0)	23.86 (15.26;38.41)**
Buses	186 (8.3)	10 (2.3)	174 (9.7)	1.20 (0.43;2.81)
Trucks	248 (11.0)	18 (4.2)	228 (12.7)	2.89 (1.49;4.51)**
Paved Roads, N (%)	2364 (95.6)	423 (96.4)	1901 (95.6)	0.41 (0.14;1.16)

(*N* number, *SD* standard deviation) *Significant at *P* < 0.05 in a bivariate association; **Significant at a *P* < 0.05 in the multivariate model; *OR* odds ratio

People involved in grievous crashes were significantly younger and were using the road at night or dawn. Grievous injuries were more associated with motorcycles and trucks as the primary crash vehicle. Primary victims with grievous injuries were mostly pedestrians and cyclists (51.8 %) or motorcyclists (32.8 %), while non-grievous crash victims were predominantly in cars (64.6 %) (Table 1).

The multivariate model found that crashes at dawn had a 58 % less chance of grievous injury than at other times of day. As the primary vehicle, motorcycles had a 4.47 times increased risk in grievous crashes; similarly, pedestrians and trucks were associated with 4.9 and 3.6 times higher risk of grievous crashes, respectively (Table 1). Pedestrians and cyclists, as the road user, had 263 times higher odds of having a grievous injury compared to car passengers. Motorcyclists and truck passengers also had increased odds of grievous injuries compared to car passengers (23.9 and 2.9 respectively) (Table 1).

**Fig. 2:**
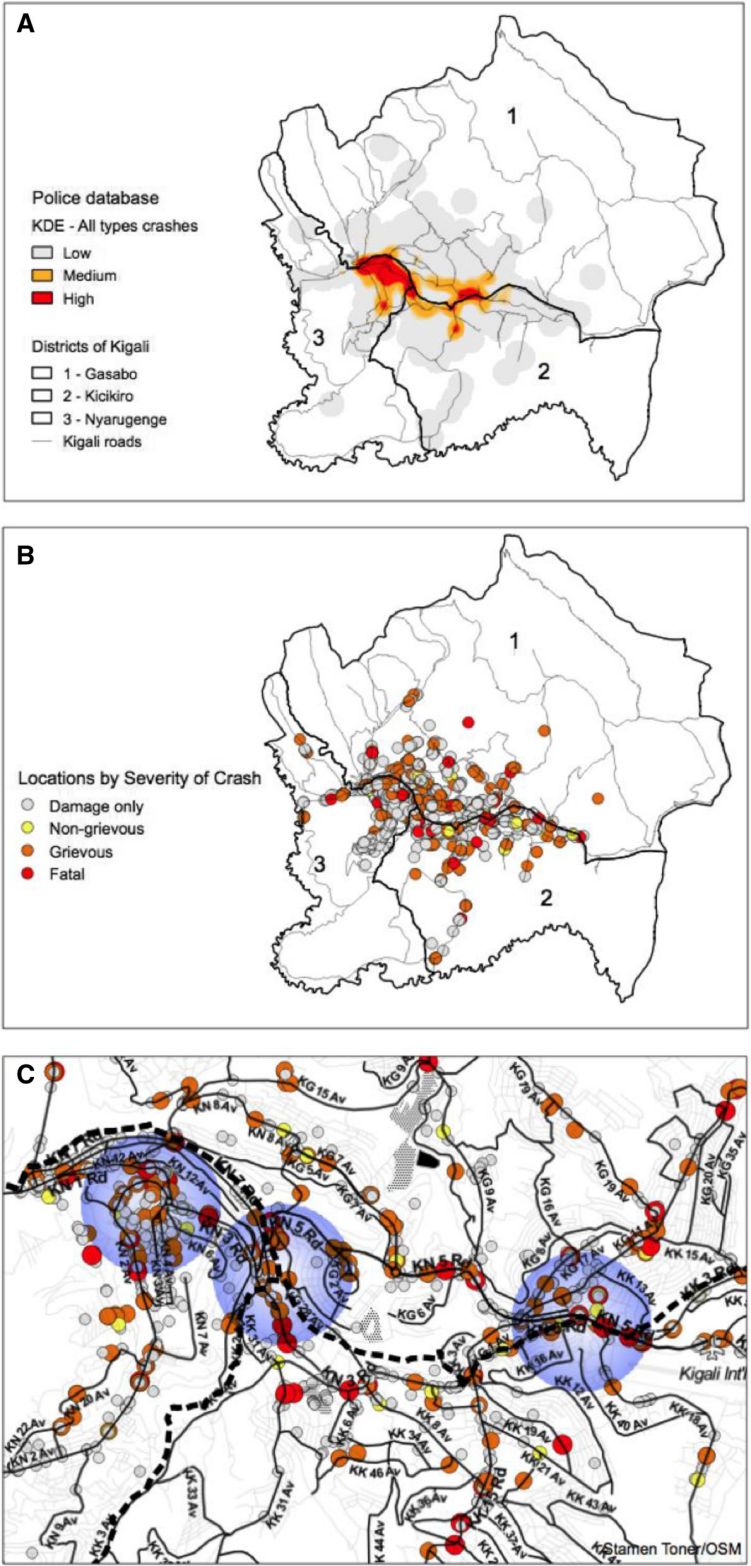
**a** Police data crashes kernel density analysis of high, medium and low density areas of all types of road traffic crashes. **b** Road traffic crash locations by severity of crash in Kigali districts. **c** Road traffic crash locations on a Kigali road map with highlighted areas of high density crashes

Of all 2589 crashes, 2212 (85.4 %) had enough information to geocode the location of the crash and therefore were included in the geospatial analysis. RTC hotspots determined by the kernel density estimator are shown in Fig. 2. RTCs investigated are mostly around the boundaries between the Gasabo, Kicukiro, and Nyarugenge districts in Kigali. Not surprisingly, the highlighted area overlaps the more densely populated area of Kigali, the Kigali City Center. High density hotspots are mostly separated in the areas overlapping the downtown areas of Kigali (Fig. 2a), on the northern region of Nyarugenge, with the boundaries of Gasabo, and the southern part of Gasabo close to the boundaries to Kicukiro. These areas are prominent in the kernel density analysis because these are the same areas with larger concentration of grievous RTCs (Fig. 2b). Gray dots are more scattered throughout Kigali compared to the red dots; this suggests that grievous RTCs are more frequent in downtown areas with higher population density and larger roads and avenues (Fig. 2c). Additionally, grievous RTC-dense areas are highlighted by blue circle on Fig. 2c.

**Fig. 3:**
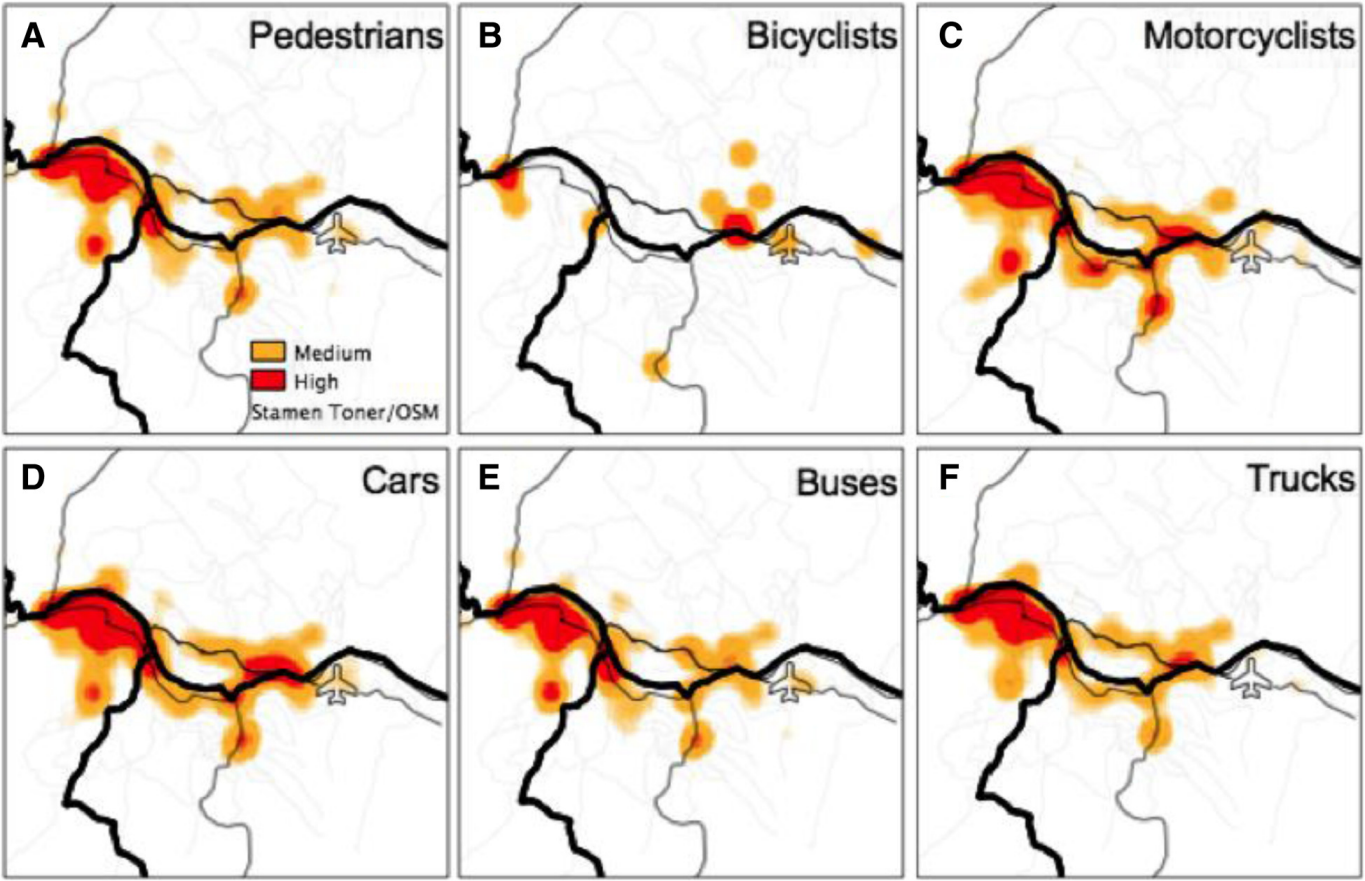
Comparison of the kernel density estimation of medium and high density hotspots of **a** pedestrians, **b** bicyclists, **c** motorcyclists, **d** cars, **e** buses, **f** trucks

**Fig. 4:**
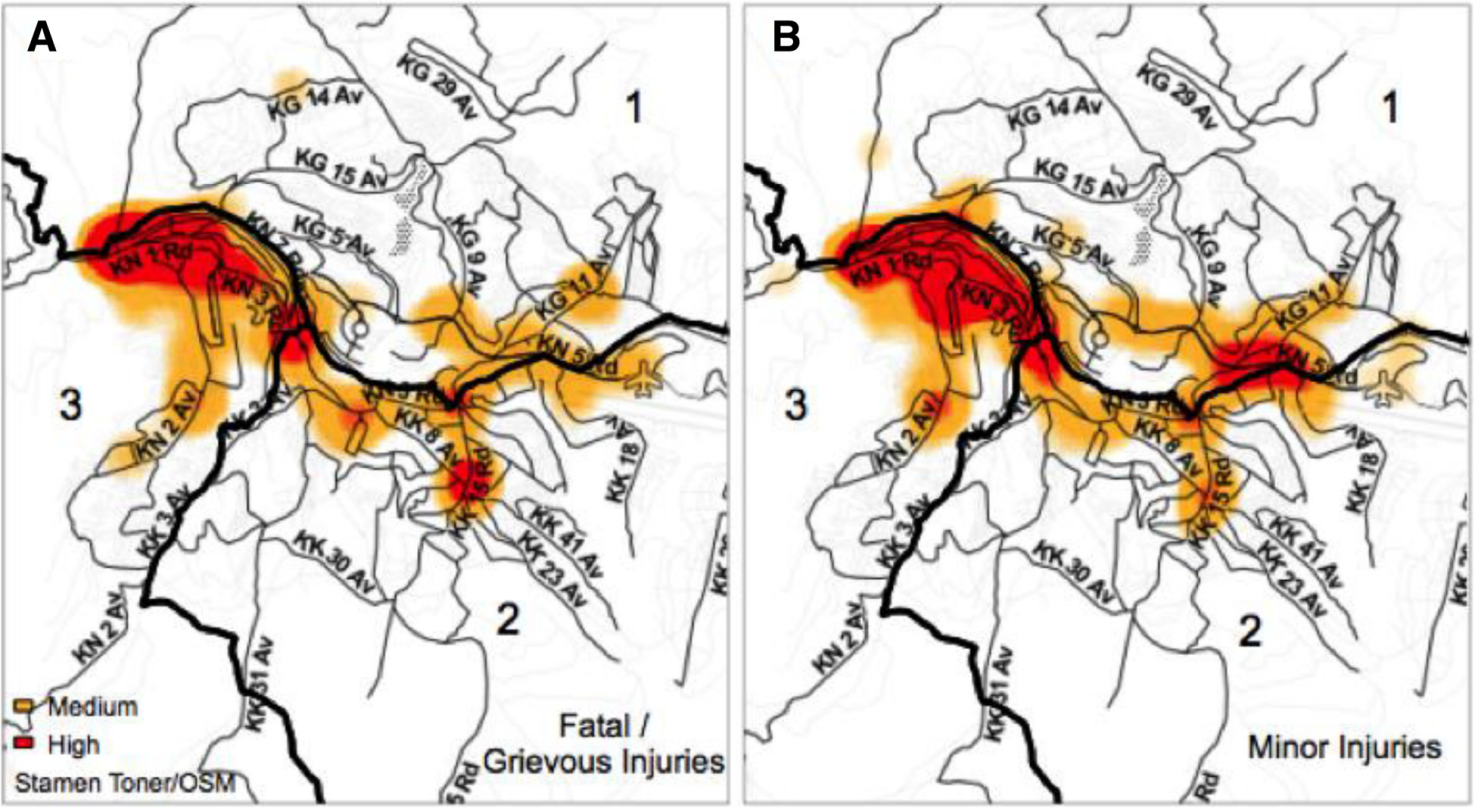
Hotspots of crashes by **a** fatal/grievous injuries and **b** minor injuries

The locations of RTC by crash severity and type of road user are highlighted in Fig. 3; and Fig. 4 highlights the hotspots for RTCs comparing minor injuries to fatal/grievous injuries. When RTCs are separated by severity (Fig. 4), the pattern of crashes is distributed similarly.

## Discussion

To our knowledge, this is the first in-depth evaluation of the epidemiology of RTCs in the Kigali region using GIS hotspot analysis from police data. Our findings demonstrated that the majority of RTC drivers were middle-aged (20 to 45 years) males. Risk of grievous injury increased with each year of decreasing age. The majority of crashes included cars and trucks, followed by buses and motorcycles. Primary victims of crashes were mainly in cars, on motorcycles, and pedestrians and cyclists. Pedestrians and cyclists, as well as motorcyclists had significantly increased odds of grievous injuries compared to car passengers.

Globally, the majority of RTCs and grievous injuries occur in young males which is also reflected in our data. While this is reflected in other studies worldwide and in Africa, but at 92.5 %, the percentage of male RTC victims in Kigali is significantly higher than worldwide estimates of 77 % [4, 18]. This is likely due to a larger percentage of male drivers in Kigali and in the country of Rwanda; there is a 10 fold higher number of licenses issued to males compared to females [10]. Despite the number of registered motorized 2- and 3-wheeled vehicles outnumbering cars in Rwanda at 43,944 compared to 40,585 cars [4], in this study we found the percentage of crashes involving by motorcycles was less than half of those caused by cars. These findings echo those from a recent systematic review evaluating road traffic deaths by user found which found that 49 % of all road traffic deaths in the World Health Organization African subregion D, which includes Rwanda, were from motorized 4-wheeled vehicles and only 16 % secondary to motorcycles [27]. In our study, although fewer RTCs were caused by motorcycles, motorcycle riders were found to have significantly higher risk of grievous injury, indicating that despite the introduction of legislature with helmet laws and limiting the number of riders on motorcycles, there should be continued focus motorcycle users and prevention of RTCs.

Our calculated numbers of injuries differed starkly from the numbers calculated in the Rwanda Statistical Yearbook from the National Institute of Statistics of Rwanda for the same year. Using police data for the country, the National Institute of Statistics determined that nationwide, the number of RTIs from motorcycles was double that of cars. Additionally, they estimated the total number of recorded injured persons from RTCs nationally in 2013 at 2609 with 315 deaths. Our data found 2589 crashes with 4687 victims, of which 685 were injured with 85 fatalities [28]. There are several possible causes for this, beginning with the large number of missing reports from the police registry. Data collection proved problematic owing to the paper reports completed by the traffic police and the limited availability of only one paper chart per traffic crash. An electronic database or production of permanent copies in a centralized database could help maintain accurate statistics. Additionally, there is a concern for inaccurate reporting at the city and/or national level police datasets, particularly given that most reports describe the majority of RTIs as occurring in Kigali. It is also possible that rural areas with fewer resources may not report RTCs as often as more centralized urban areas. Although previous studies have evaluated rates of underreporting RTCs in more urban areas, limited work has been done in rural areas, and none that we know of in Rwanda [29, 30]. Underreporting of data on RTIs is not only limited to Rwanda, but is a problem throughout Africa and other LMICs [30–34]. The WHO suggests linking of data sources such as police, insurance, and registration records to improve data on road traffic deaths, however only a handful of primarily high-income countries use this method to determine official fatality rates. Data on non-fatal RTIs is even more disparate owing to variations of definitions and data collection methods [35]. In Rwanda, a quality analysis of police data in addition to surveys of the community could yield additional information in determining discrepancies in data or reasons for underreporting of data by the public.

Geospatial analysis demonstrated that most of the hotspots are clustered around the intersection of the three districts of Kigali Province and seem to follow 2 major highways that traverse the city, RN3 and RN15, in addition to roads KN3 and KN5, near the Kigali International Airport. When separated by severity of injury, grievous injuries are noted to occur more in larger roads or highways, intersections, and densely populated areas of Kigali. This is likely due to increased speed on the highway and larger roads with an increased pedestrian and vulnerable road user density. Considering these distinct hotspots and the WHO literature suggesting road safety audits, the next area of research should be conducting a built environment analysis at these locations to identify more specific causes of risk for road users [4].

It has been suggested that many of the problems leading to RTIs in Sub-Saharan Africa center around politics, cost, resources, and enforcement [36]. The Rwandan government made vast improvements to infrastructure and began implementing road safety programs following the genocide in the 90s. The most significant changes included seatbelt laws, vehicle inspections, and speed limits in addition to overhauling law enforcement by cracking down on police corruption and increasing police resources. Additionally, Rwanda was one of the first African countries to implement helmet laws and to limit the number of riders on motorcycles. These changes led to significant overall reductions in mortality from RTIs, including markedly lower rates of motorcycle crashes compared to other LMIC countries but rates of RTIs still remain significantly higher compared to Western countries [4, 37]. The goal of our project was to not only conduct a hotspot analysis which will provide the platform for conducting a built environment analysis in Kigali Rwanda, but to evaluate the quality of police data in Rwanda for this methods. Most LMIC have limited quality so this method is challenging. The next steps for this project in Rwanda are to conduct the built environment analyses at our stated hotspots as well as safety program planning. Similarly, further investigation and initiatives specifically for VRUs like motorcyclists and pedestrians are needed, particularly because of their risk of grievous injury. Even with the obvious data quality challenges, given the high rates of geolocation of crash sites from the police data, Rwanda could serve as a model for other LMIC police data collection.

### Limitations

The RTC reports analyzed in this study were obtained from the Kigali Traffic Police and therefore subject to the completeness of their database. The traffic reports consist of single paper copies, therefore they were unavailable if an RTC was not reported, or if it was being investigated or processed by the courts or insurance providers. Of note, grievous RTCs and those resulting in substantial property damage are anecdotally reported to make up the majority of RTCs that are brought to the judicial system. Once a report is sent to the judicial system it effectively disappears from Kigali Traffic Police and is never returned. This limitation could substantially impact the ability of our results to reflect the true nature of RTC hotspots in Kigali and the true number of grievous versus non-grievous RTIs in this study. There was no way to verify the suggested non-random nature of missing information due to the limited nature of those missing sheets or the register log. Similarly, missing data was present in our overall cohort; most notably, we were not able to identify geolocation of 14.6 % of our data. Road conditions were also missing and some data was likely missing in our overall cohort. Previous studies have demonstrated underreporting of RTCs to police in LMICs [16, 30]. Future studies could involve combining information from hospital records and insurance claims for a more complete representation of RTCs. Additionally, given that the injury severity is subjectively determined by police officers rather than trained healthcare personnel, the extent of grievous injuries may be limited. Although we were able to obtain information on the numbers of new licenses issued per year by gender, we were unable to find data regarding the ages of licensed individuals. This limits our ability to compare injury severity in the context of licensed individuals.

While the best approach to understand determinants for road traffic injury hotspots would be to control for exposure in each location, in a LMIC setting such data is not always readily available. Limitations with the quality of data, volume of road users, and commuting characteristics restrict our ability to fully evaluate these hotspots. A geospatial analysis based on areas (instead of points) could be used to control for population, but this was not the goal of the paper. Instead, our goal was a more commonly used point distribution analysis for identifying hotspots. Although we did not control for exposure, our methodologic approach was able to identify the locations with the highest density of crashes. Future research will focus on these variables in order to further characterize the built environment and determine potential changes to reduce crashes.

## Conclusion

This study of the epidemiology of road traffic crashes in Kigali, Rwanda echoes what is widely known regarding road traffic injuries in low and middle income countries; young males are disproportionately involved in road traffic crashes and vulnerable road users (motorcycle drivers, cyclists, and pedestrians) are more likely to incur grievous injuries from road traffic crashes. With our use of geospatial analysis, we were able to identify specific hotspots which might be amenable to intervention. Next steps include in depth evaluations of these locations so local and central governments can in implement appropriate road safety measures. Our project also highlights the need for more complete and higher quality dataset and an improved reporting system of road traffic crashes. Simple improvements in data collection could potentially improve rates of road traffic injuries by allowing for more accurate analysis of determining user-specific and built environment causes of crashes.

## Abbreviations

EDSA, exploratory spatial data analysis; GIS, geographic information systems; LMIC, low- and middle-income country; MI, multiple imputation; REDCap, Research Electronic Data Capture; RTC, road traffic crash; RTI, road traffic injury; VRU, vulnerable road user
